# Quantile connectedness in agri-commodity markets: What has changed over past six decades?

**DOI:** 10.1016/j.heliyon.2023.e13463

**Published:** 2023-02-08

**Authors:** Bikramaditya Ghosh, Dimitrios Paparas

**Affiliations:** a Symbiosis Institute of Business Management, Symbiosis International, Deemed University, Bengaluru, 560100, India; b FLAM Department, Harper Adams University, Newport, TF10 8NB, UK

**Keywords:** Commodity markets, Quantile VAR, Risk spillover

## Abstract

Agri commodities have been investigated in the past to determine their inter-relationships. However, no study has checked their risk spillover/connectedness for six decades using extreme quantiles. Various shocks (positive/negative) often pose challenges to these commodities over the past six decades. Such shocks’ impact is usually observed in extreme quantiles or tails. Therefore, we have investigated fourteen agri commodities (namely Coffee, Cocoa, Soyabean, Wheat, Sugar, Orange, Chicken, Beef, Maize, Tea, Coconut Oil, Groundnut Oil, Palm Oil & Rice) from January 1, 1960 to June 1, 2022 (covering 62 years on a monthly basis), deploying Quantile VAR or QVAR as suggested by [1](extended [2,3] calibration). We found that the risk spillover/connectedness never came down for these Agri commodities. It is always at a higher level (more than 55%) proving that agri commodities remain vulnerable to various shocks throughout. Spillover looks symmetric as both the extreme tails enjoy about 92–93% connectedness levels, whereas the median is below 60%. Rice, Orange Juice, Chicken, Tea and Groundnut Oil were consistent net receivers across such a long-time frame, whereas Palm Oil, Soyabeans, Maize and Wheat were net emitters all through. Further, we found decreasing complexity (network connectedness reduction) with increased quantiles. Since these findings are over such an extended period, policy decisions can be made based on them.

## 1 Introduction

It has been observed that most agri-commodities such as Wheat, corn, and soybean enjoy cyclical trajectory. Historically their prices began surging in 1971 till 1974. However, it declined afterward, albeit slowly, and steadied at a level above the 1960s. Crop prices experienced another surge in 1990 and continued to increase until 1994, reaching their highest point in 1995 for corn and wheat, and 1996 for soybeans, before experiencing a significant decrease. The Soviet Union's large purchase of grain in the global market in the early 1970s was a significant factor in stimulating global demand for crops. Countries that are centrally planned followed suit, fuelling the grain imports and helping the prices rise significantly (almost 30% by global standards). This was coupled by an abundance of petrodollars by major oil-exporting countries; the 1970s witnessed nearly 5% growth in global agricultural commodity imports [4].

The transition of the U.S. dollar from the gold standard to a floating exchange rate caused a persistent devaluation of the currency against major currencies, with the dollar losing nearly 30% by the 1980s. Additionally, the failure of Peruvian anchovy catches in 1972 led to a shortage of high-protein feedstocks and an increase in demand for soybean meal, causing soybean prices to rise in 1973 and 1974. The 1990s witnessed the growth of newly industrialized Asian counterparts. This led to the hike of agri-commodity prices between 1993 and 1996. A shortage of food grains further fuelled it due to below-normal harvesting. US, too reduced carryover stocks to support prices. However, it ended abruptly owing to financial crisis in 1997–99. The mid-2000s saw a rapid increase in global food grain demand (up by 50% between 2000 and 2006). This was, however, due to the rise in income in developing nations (accounting for 63% of total US agri exports). Further those developing nations witnessed diversification in consumer diets (more vegetable oil, meat and dairy produce) in the 2000s. Grains and livestock feed seeds rose disproportionately from 2000 to 2008 [4]. Typically, prices of agricultural commodities increase economic and social costs. In addition to that, it may also affect health and education. Intuitively, some commodities transmit shock whereas others receive it. Moreover, various agricultural commodities’ interdependence and risk transmission vary with time. Sometimes the changes are symmetric; otherwise asymmetric primarily due to legislation in the farm sector [5]. A stressed effect usually increases the total connectedness (TCI) in a network. Further, the TCI varies drastically across quantiles [6,7]. Of late many studies have been carried out between agri commodities and oil shocks, industrial & energy prices, metal markets etc. [[8], [9], [10], [11], [12]]. They dealt with various shocks towards agri commodities from other assets during stressful situations such as Covid. The common thread being the investigation on the agri commodities over a very long period of time. Most of these works used the same methodology or at least somewhat similar methods, which is used by us. Having said that, wavelet methods and various Copula methods would be an alternative as well. This confirms the solidity and robustness of our methodology over a long period. They too found significant volatility connectedness among agri commodities especially during stressed periods. They also find monetary policy to be influencing agri commodities by quite a significant way [13]. They have even pointed out to an option of using agri commodity as a dynamic hedge in a multi-commodity portfolio [8,14]. In fact, we have considered many such literature to finalise the agri commodities under consideration for our investigation.

However, no study has been found to date on Agri commodities along these lines. Therefore, a definite research gap exists. Identifying this kind of spillover mechanism is crucial for policymaking regarding Agri commodities.

The idea for this project came from a relatively older research paper from US Catfish markets, where EGARCH showed strong risk transmission from the feeding materials to the Catfish prices [15]. However, this study requires complex mechanisms such a TVP-VAR or Quantile VAR. Therefore, the statistical tools that got developed between 2012 and 2021 [7,[16], [17], [18]]would be of good use to detect such kind of risk transmission.

We have several objectives behind this attempt.

1. To understand the connectedness/spillover between various agri commodities over time
2. To identify the risk (shock) emitters and receivers among agri commodities
3. To check the impact of stressed events on agri commodities globally during the past decade or so
4. Study and identify the type of connectedness (symmetric or asymmetric) across extreme quantiles
5. To identify the embedded stylized facts during this investigation

After the introduction and a review of existing research on the topic, the next part offers information on the research method and data applied. The third section presents the results of the analysis. The fourth section examines the results in more detail. Lastly, the fifth section suggests policy recommendations.

## 2 Data & research methodology

We have selected fourteen Agri-commodities return series (namely Coffee, Cocoa, Soyabean, Wheat, Sugar, Orange, Chicken, Beef, Maize, Tea, Coconut Oil, Groundnut Oil, Palm Oil & Rice) from World Bank Commodity Price Data (The Pink Sheet) from January 1, 1960 to June 1, 2022 (covering 62 years on a monthly basis). We've considered log returns since they are more stochastic in nature and follow the zero mean and constant variance more closely than the closing price data.

Firstly, ample literature needs to be referred along these lines. Secondly, we need to justify each objective with existing literature. Thirdly, sourcing the data (from credible sources such as The World Bank) and processing it through Quantile Connectedness calibration (Quantile-VAR or Quantile Vector Auto Regression) suggested by Refs. [[18], [19], [20]]. Lastly, we need to decipher the results and formulate strategies for the policymakers, especially during stressed events. We have considered a window size of 200, as suggested by some recent research work [18,21]; the window size strongly influences connectedness. A small window increases the volatility and a large one level the same. We will measure asymmetry/symmetry in extreme quantiles (as suggested by existing literature) by deploying Quantile-VAR. We will check the TCI movement before, after and during each of the stressed events. In addition to that, we will check the network plot to identify the risk (shock) emitters and receivers. Our approach will be strictly quantitative. However, we will try to build specific ‘stylized facts’ for agri commodities.

Stylized facts are typically born out of large empirical analysis in a specific domain and remain consistent observations with intuitive reasoning for years to come. British mathematician Rama Cont developed the idea of stylized facts back in early 2000 [22]. It has been gaining strength from hence on. It assists the policymakers in deciding on certain aspects based on a consistent pattern of a stochastic time series. Since all the agri commodities are fundamentally represented by stochastic time series [23], therefore, it would be rather useful to find such stylized facts. Stylized facts are common observations, mainly on economic/financial time series, which are consistent over long periods. These are usually observed when extensive data processing and modeling is conducted over a long period. They start as mere observations and ultimately become theory. Autocorrelation, long range dependence, fractional movement, volume-volatility relationship is some of the popular ‘stylized facts’ in economics and finance [24].

Our method modifies the connectedness context by Refs. [2,3]. Following [25], we apply quantile vector autoregression to examine the connectedness between fourteen Agri-commodities monthly log return series (namely Coffee, Cocoa, Soyabean, Wheat, Sugar, Orange, Chicken, Beef, Maize, Tea, Coconut Oil, Groundnut Oil, Palm Oil & Rice) from January 1, 1960 to June 1, 2022 between the extreme lower, median, and extreme upper quantiles. This method permits us to take into account the extreme market movements throughout countless extreme event across the past 62 years. The estimation of quantile vector autoregression, QVAR(p) is:

(1) $$y_{t}\left(\tau \right)=\mu \left(\tau \right)+\sum_{j=1}^{p}\varphi_{j}\left(\tau \right)\;y_{t-j}+u_{t}\left(\tau \right)$$

Where, t denotes time, τ denotes the quantiles; $y_{t}$ is a vector of n endogenous variables, including fourteen Agri-commodities $\mu \left(\tau \right);\;\varphi_{j}\left(\tau \right)$ represent coefficient matrices while $u_{t}\left(\tau \right)$ denotes the error vector. The maximum lag length orpis4; which is as per existing literature from Ref. [26] as well as [[27], [28], [29]]. Applying Wold's theorem, we modify the QVAR(p) in equation (1) to a quantile vector moving average representation, QVMA(∞): $Q_{\tau }\;\left(y_{t}|F_{t-1}\right)=\mu \left(\tau \right)+\sum_{i=0}^{\infty }A_{i}\left(\tau \right)u_{t-i}\left(\tau \right)$, with $A_{i}\left(\tau \right)=\Theta_{1}\left(\tau \right)A_{i-1}\left(\tau \right)+\Theta_{2}\left(\tau \right)A_{i-2}\left(\tau \right)+...$ for $i=1,2,....\;;\;A_{0}\left(\tau \right)=I_{n}$ and $A_{i}\left(\tau \right)=0$ for i<0. $I_{n}$ is an n×n identity matrix. We use the QVMA(∞) representation and we calculate the H-step ahead generalized forecast error variance decomposition (GFEVD) as equation (2):

(2) $$\psi_{i,j,\tau }^{g}\left(H\right)=\frac{\sigma_{jj}^{-1}\sum_{h=0}^{H-1}{\left(e_{i}^{T}A_{h}\left(\tau \right)\Sigma e_{j}\right)}^{2}}{\sum_{h=0}^{H-1}\left(e_{i}^{T}A_{h}\left(\tau \right)\Sigma A_{h}{\left(\tau \right)}^{T}e_{i}\right)}$$

Σ is the variance matrix of the error term vector; $\sigma_{jj}$ signifies the standard deviation of the error term of j. $e_{i}$ is a n×1 vector that takes the value 1 for element i and 0 otherwise. Afterward, we calculate the normalized Generalized Forecast Error Variance Decomposition (GFEVD) [30,31]. GFEVD is employed in this methodology as a measure of robustness (equation (3)).

(3) $${\psi ͂}_{ij,\tau }^{g}\left(H\right)=\frac{\psi_{ij,\tau }^{g}\left(H\right)}{\sum_{j=1}^{k}\varphi_{ij,\tau }^{g}}$$

${\psi ͂}_{ij,\tau }^{g}$ (H) shows the percentage of forecast error variance in i that is described by j when i is in quantile τ. Subsequent, we compute the next spillover indexes to take the overall spillovers among the variables:

(4) $${FROM}_{i,\tau }\left(H\right)=\frac{\sum_{j=1,j\neq i}^{n}\psi_{ij,\tau }^{g}\left(H\right)}{n}\times 100$$

(5) $${TO}_{i,\tau }\left(H\right)=\frac{\sum_{j=1,j\neq i}^{n}\psi_{ji,\tau }^{g}\left(H\right)}{n}\times 100$$

(6) $${NET}_{i,\tau }\left(H\right){=TO}_{i,\tau }\left(H\right)-{FROM}_{i,\tau }\left(H\right)$$

(7) $${TCI}_{\tau }\left(H\right)=\frac{\sum_{i,j=1,j\neq i}^{n}\psi_{ji,\tau }^{g}\left(H\right)}{n}\times 100$$

The TO connectedness index specifies (equation (4)) the overall impact i has on all other variables j. The FROM connectedness index (equation (5)) demonstrates the effect of shocking all other variables j on i. The NET connectedness index (equation (6)) gets the net spillovers from i to all other variables j, where a positive (negative) value show that i is a shock transmitter (receiver) in the system. Lastly, the total connectedness index (TCI) (equation (7)) is take the overall connectedness between the variables in the system and is used a proxy for market risk contagion.

In the empirical examination, we emphasize on verifying the quantile connectedness at the 0.1, 0.5 and 0.9 quantiles. Those quantiles captures the interconnectedness between 14 agricultural commodities during extreme negative, median, and extreme positive movements. Additionally, to the static interconnectedness, we examine the dynamic interconnectedness by estimating the rolling spillover indexes using a rolling window of 200 days.

The methodology adopted here have certain clear advantages over its peer group models such as TVP-VAR and DCC-GARCH. Primarily all these models are extensions of [2,3] connectedness model. In QVAR [1] the interaction and feedback effects among the variables dependent on their quantile dynamics. Since, shocks (positive/negative) have visible effects on extreme quantiles, this methodology is apt for this kind of study.

## 3 Empirical results

**Fig. 1:**
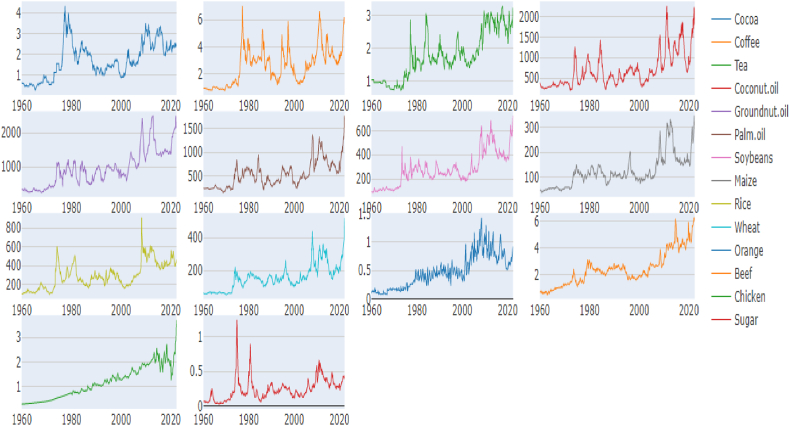
Agri commodity time-series over the years

**Figure 1.1:**
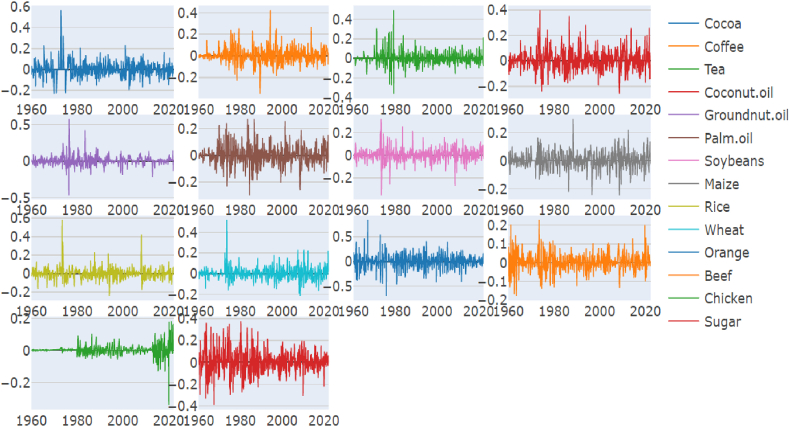
Agri commodity log returns over the years.

In general, prices of most of agricultural commodities such as Cocoa, Coffee, Tea, Coconut oil, groundnut oil, palm oil, soyabeans, maize, rice, wheat, and sugar (Fig. 1, Fig. 1.1) were relatively stable in the 1960s, but began to rise in the 1970s and reached a peak in the late 1970s and early 1980s. After that, prices dropped significantly and remained low during the 1990s and early 2000s. Prices then started to increase again, reaching a peak in the late 2000s and early 2010s. Since then, prices have fluctuated but overall, the trend for most of the commodities is that prices have been increasing over the time. Other commodities such as orange, beef, chicken were relatively stable between 1960 and 1980, and then prices have been increasing. The prices of the commodities can vary depending on a number of factors such as weather, supply and demand, and government policies.

We have some interesting general findings before addressing our specific objectives for this study. To begin with, we find that Coconut oil, Palm Oil, Maize, Beef and Sugar are platykurtic, therefore do not have fat tails (See Table 1). This means that their price predictions will not fluctuate a lot and are predictable by and large with a fair bit of accuracy. While the other commodities under consideration have wide fluctuations and fat tails, therefore they are leptokurtic. Periods having financial crisis usually generate extensive connectedness between commodities. Past studies have proved it for Crude Oil and Gold ([32]). Therefore, our study has addressed the research gap. The next part of this section will address our specific objectives thorough the empirical observations.

**Table 1:** Descriptive statistics.

	Cocoa	Coffee	Tea	Coconut oil	Groundnut oil	Palm oil	Soybeans	Maize	Rice	Wheat	Orange	Beef	Chicken	Sugar
Mean	0.002	0.002	0.001	0.002	0.003	0.003	0.003	0.003	0.002	0.003	0.003	0.003	0.003	0.003
Variance	0.004	0.005	0.004	0.006	0.004	0.005	0.003	0.003	0.003	0.003	0.016	0.002	0.001	0.01
Skewness	1.04***	0.640***	0.984***	0.412***	0.917***	0.058	0.069	−0.008	2.070***	1.253***	−0.029	0.156*	−0.86***	0.203**
	0.0	0.0	0.0	0.0	0.0	0.0	0.0	0.0	0.0	0.0	0.0	0.0	0.0	0.0
Kurtosis	8.9***	4.37***	11.13***	1.896***	17.3***	2.2***	6.3***	2.9***	18.1***	10.55***	5.41***	2.52***	22.01***	1.62***
	0.0	−0.8	0.0	0.0	−0.1	0.0	0.0	0.0	0.0	0.0	0.0	−0.1	0.0	0.0
JB	29.5***	42.4**	44.5***	304***	82.3***	259***	92.6***	326***	76.3***	243***	47.5***	86.6***	37.9***	897***
	0.0	0.0	0.0	0.0	0.0	0.0	0.0	0.0	0.0	0.0	0.0	0.0	0.0	0.0
ERS	−1.2	−1.4	−0.8	−2.3	−0.6	−0.5	0.0	−0.3	−1.4	0.5	−1.2	1.1	1.8	−2.4**
	−0.2	−0.2	−0.4	0.0	−0.5	−0.6	−1.0	−0.7	−0.2	−0.6	−0.2	−0.3	−0.1	0.0
Q (10)	374***	3322**	3698**	3235***	3649***	3251***	3500***	3456**	3487***	3327***	3427**	3715**	3689**	3082**
	0.0	0.0	0.0	0.0	0.0	0.0	0.0	0.0	0.0	0.0	0.0	0.0	0.0	0.0
Q^2^(10)	351***	2816**	3558**	2811***	3417***	2683***	3188***	3126**	3007***	2707***	2933**	3453**	3085**	2077**
	0.0	0.0	0.0	0.0	0.0	0.0	0.0	0.0	0.0	0.0	0.0	0.0	0.0	0.0

JB:Jarque-Bera test statistics, ERS: Elliott, Rothenberg & Stock Unit Root test statistics. Q (10) and Q^2^(10): Ljung-Box test statistics on returns and squared returns. ***, **, * indocate statistical significance at 1, 5, and 10% level.

**Fig. 2:**
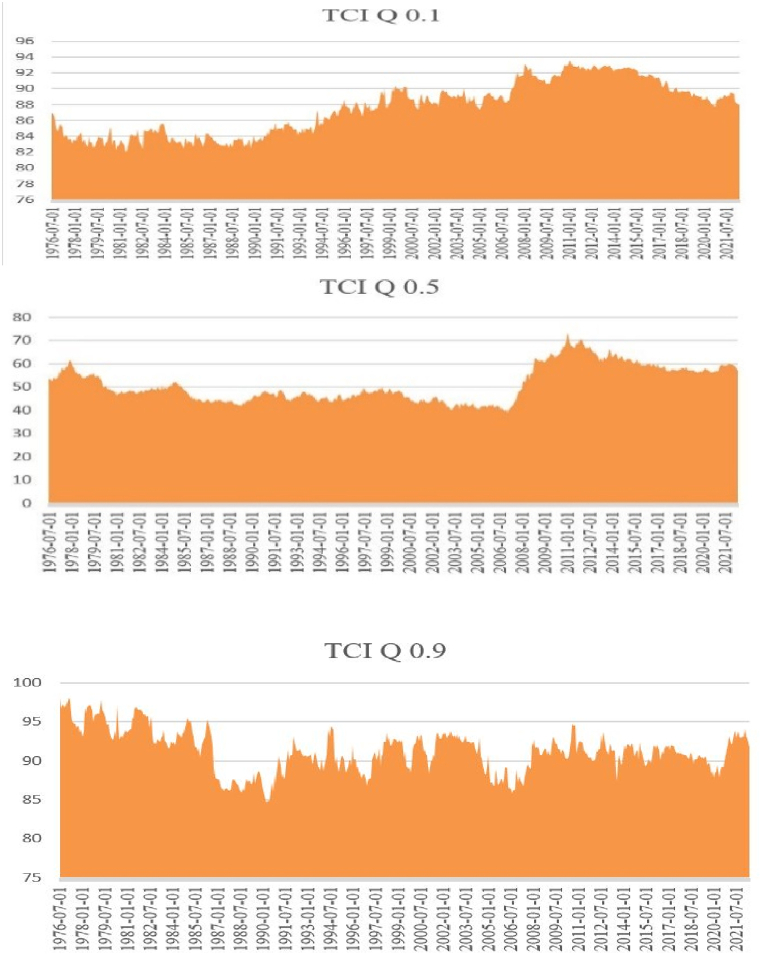
TCI over the years (TCI 0.1, TCI 0.5 & TCI 0.9 Quantiles).

**Fig. 3:**
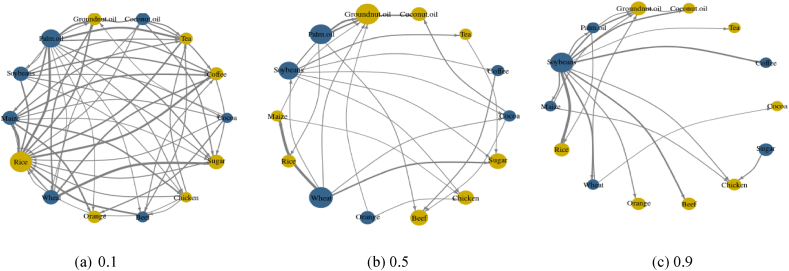
Network Plot (Blue represents net-emitter, whereas yellow represents net-receiver; 0.1, 0.5 & 0.9 Quantiles). Note: The figure displays a network graph depicting the interconnections between the agricultural commodity across different quantiles. Blue (yellow) nodes imply net shock transmitters (receivers) and the size of the nodes relates to the absolute values of the NET connectedness index. The direction of the arrows illustrates the direction of spillovers among two variables, and the thickness of the arrows implies the intensity of these spillovers. (For interpretation of the references to colour in this figure legend, the reader is referred to the Web version of this article.)

Our results showcased the time-varying connectedness/spillover between various agri commodities, satisfying our first objective (See Fig. 2). Rice, Orange, Chicken, Tea & Groundnut Oil were net receivers consistently across extreme quantiles (See Fig. 3); Palm Oil, Soyabean, Maize and Wheat were consistent net emitters in extreme quantiles as well. Interestingly complexity of spillover/connectedness among these commodities reduces drastically as they approach extremely higher quantile (i.e., 0.9). These observations satisfy our second objective as well.

**Fig. 4:**
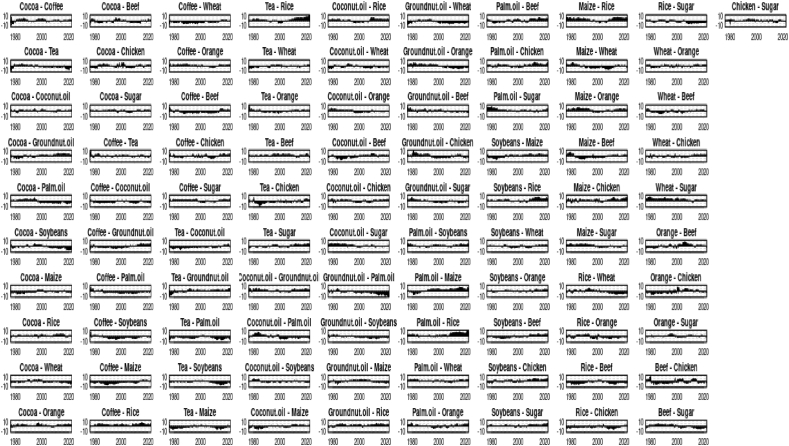
Net pairwise directional connectedness (Q- 0.1)

**Fig. 4.1:**
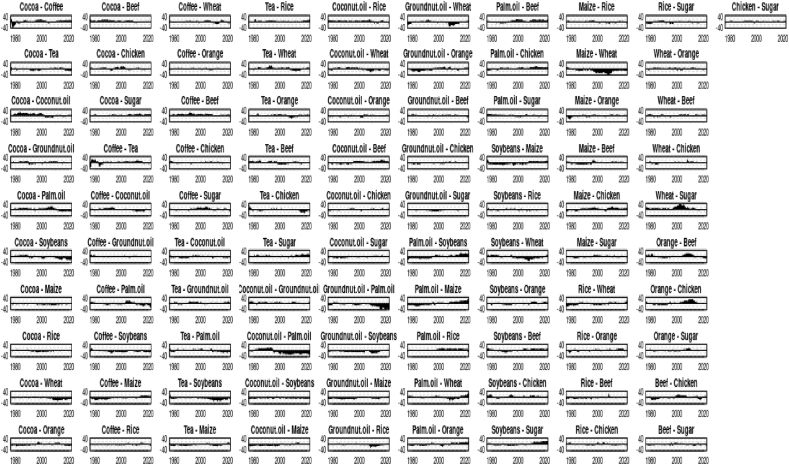
Net pairwise directional connectedness (Q- 0.5)

**Fig. 4.2:**
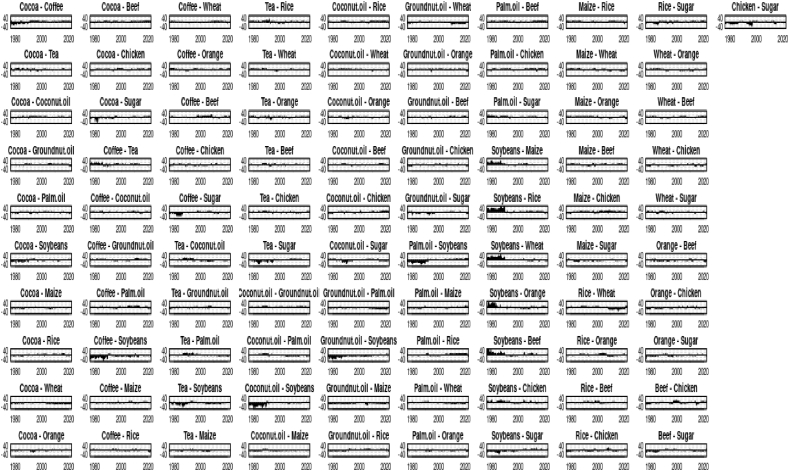
Net pairwise directional connectedness (Q- 0.9).

**Fig. 5:**
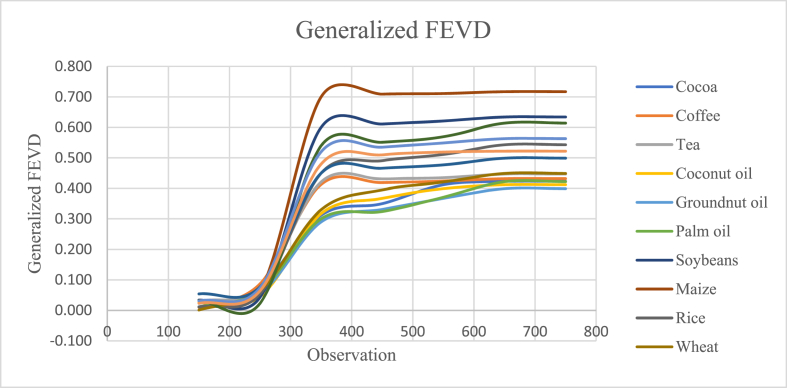
Generalized Forecast Error Variance Decomposition (GFEVD) for all the commodities through QVAR.

Moving onto our third objective of finding impact of stressed events on connectedness we found that agri commodities are no exception to this rule. We found TCI (depicting connectedness) has a minimum value of 83–85% for both the extreme Quantiles i.e., 0.1 & 0.9 (See Fig. 2). Uncertainty in many areas and demand-supply mismatch owing to war like situations (for past 62 years) fuelled the price volatility of these commodities. Sometimes, the impact is not direct, however, the impact is due to disruption of the global and domestic supply chain [33].

The fourth objective searched for symmetric and asymmetric connectedness. It has been proved that median quantile is not an accurate depiction of connectedness, unlike both extreme quantiles, which agrees with a current study [34]. Therefore, we emphasize more on the two extreme quantiles. Asymmetric connectedness has been observed here. TCI is usually used as a proxy for market risk [20] with its higher value indicating higher interconnectedness. Six decades (62 years precisely) of global agri-commodities with the lowest terminal TCI (at 0.1 and 0.9 quantiles) of 83–85% signifies the inherent market risk in the global agri-commodity market. Therefore, the observation does satisfy our fourth objective.

The fifth and last objective is to find the presence of ‘stylized facts’ if any. We found a substantial reduced complexity inside the network of emitting and receiving shocks (See Fig. 3a,b,3c) at a significantly higher quantile. This can be a new ‘stylized fact’. Therefore, all our objectives have been fulfilled. Since this methodology is novel and proven [18,35] and no other study period has been this vast and inclusive in nature, therefore this study would most certainly add incremental knowledge to the existing literature.

All our calibration is in perfect accordance with the typical shape of GFEVD curve and the value range specified (0.00–1.00), depicting that the errors are well within control. Our results are consistent with the existing literature on this error plotting [36].

## 4 Discussion

The connectedness or risk spillover between major agri commodities is relatively unexplored. Although, it can produce a significant outcome and assist policymakers if appropriately explored. The stylized facts that emerged from our study would certainly be the incremental contribution to the existing literature. Our study outcome has been entirely consistent with certain other studies. First, we found the extreme quantiles (0.1 & 0.9) being highly connected for past 62 years. This means that the crisis of Agri-commodities never reduced over the years (See Fig. 2). This observation can be attributed to several factors. Structural changes such as rapid industrialization (led by manufacturing & services), a reduction in agriculture, and an increase in population almost together never allowed this sector to settle down [37]. In addition to that resources depleting process (such as mining and deforestation) cobbled with a sustained inflow of international aid would undoubtedly diminish the agricultural comparative advantage of any country [38,39]. The supermarket revolution over the years, accompanied by changes in the food value chain and abrupt changes of agricultural incentives, also led to uncertainty [37,40]. Climate change, agri research and development driven policy changes fuelled the uncertainty in this sector further [41,42]. Second, Palm Oil, Soyabean, Maize and Wheat were found to be net emitters in extreme quantiles (0.1 & 0.9) (see Fig. 3) which is consistent with the findings of the latest work [9,43]. We found, Soyabeans to be connected with most agri commodities (8 out of 14 commodities). Soyabeans accounted for 10–14% of returns in Maize, Rice, Beef, Wheat, Oils (Palm, Groundnut & Coconut), and coffee (See Table 2c) during extreme high quantile. This clearly indicates uncertainty spillover due to stressed events globally. Our data being large have witnessed various stressed events with far-reaching consequences over last 62 years such as Vietnam war, Bangladesh war, Iraq war, USSR collapse etc. Another exponent which is net emitter in extreme quantiles, Soyabean (see Fig. 3A,B & C) has witnessed excess consumption in last 50 years [44]. Third, we find a rather interesting outcome. Maize, Soyabean and Sugar are transmitting shocks to Chicken at 0.9 (extreme) quantile (see Fig. 3C). While investigating the matter we found evidence of Sugar being the new dietary supplement for Chickens alongside soyabean and maize [45,46]; in addition to that soyabean and maize are the tested and proven poultry feed [47]. Fourth, we found the contributions to others (To) and from others (From) is stronger in both the extreme quantiles than that of the middle (See Table 2a, Table 2ba–2b), which is consistent with another research involving other asset classes ([[48], [49], [50]]). Fifth, Rice, Orange, Chicken, Tea & Groundnut Oil were net receivers in extreme quantiles (0.1 & 0.9) (Fig. 3), Rice has been proven as an unstable market, with an uncertain supply and highly volatile prices, therefore it is intuitively an absorber of external shocks [51]. The pass-through price elasticity of chicken has been relatively high, therefore making it an eligible commodity as a shock-receiver [52]. Orange, globally traded as frozen concentrated orange juice (FCOJ) has a proven track record of reacting to ‘supply shocks’ [53]. IMF research has found that shocks to Groundnut Oil and Tea are not persistent consistently, albeit it would take a long time to dissipate [54]. Sixth, as quantiles increased complexity of the network connectedness reduced (from 0.1 to 0.9.) (Fig. 3). We have found similar observation and consistency in stylized facts with another research work. This proves that network connectedness at the lower quantile is higher than that at the upper and median quantiles [55]. Chaos (inter-connectedness) inside the network came down substantially with higher to extreme quantiles. All agri-commodities were giving and receiving shocks at Q = 0.1, whereas select are receiving/emitting shocks at Q = 0.9 (Figs. 4, 4.1, 4.2).

**Table 2a:** Average Dynamic Connectedness (using QVAR having 0.1 Quantile).

	Cocoa	Coffee	Tea	Coconut oil	Groundnut oil	Palm oil	Soybeans	Maize	Rice	Wheat	Orange	Beef	Chicken	Sugar	FROM
Cocoa	22	6.66	6.96	6.13	5.66	7.14	7.1	6.21	3.82	5.96	5.01	6.63	5.81	4.93	78
Coffee	7.43	21.33	6.82	6.49	5.7	7.25	7.43	7.16	3.06	6.92	4.45	6.19	4.7	5.07	78.67
Tea	6.2	7.17	19.83	7.24	5.31	8.16	7.09	7.15	4.34	4.78	6.32	5.42	6.47	4.52	80.17
Coconut oil	6.55	4.91	5.66	16.39	6.05	10.74	7.48	7.57	5.09	7.75	5.27	6.67	5.61	4.26	83.61
Groundnut oil	6.51	5.35	5.78	7.59	15.92	9.41	7.49	6.62	4.33	7.08	5.31	6.01	6.23	6.37	84.08
Palm Oil	6.65	5.59	5.51	10.17	7.06	15.8	8.64	7.03	4.63	7.76	4.75	6	5.23	5.18	84.2
Soybeans	5.85	5.56	5.62	7.37	6.41	9.67	14.78	9.55	3.91	8.72	4.44	6.97	5.8	5.33	85.22
Maize	4.97	5.01	5.59	7.37	6.58	9.02	9.97	14.4	4.27	10.71	4.23	7.16	4.81	5.91	85.6
Rice	5	5.79	6.44	6.29	6.31	7.56	5.88	7.3	17.01	6.49	7.08	7.29	5.14	6.42	82.99
Wheat	5.24	4.92	4.34	6.7	5.3	8.65	8.62	9.71	5.5	17.99	5.31	6.84	5.11	5.77	82.01
Orange	6.61	5.23	6.42	5.39	5.32	5.65	4.99	6.29	6.17	5.39	23.17	6.76	7.41	5.19	76.83
Beef	6.09	4.97	6.76	6.21	6.28	6.96	6.79	6.62	4.96	6.21	7.4	18	6.86	5.88	82
Chicken	5.82	4.58	5.17	6.17	6.54	6.45	6.85	6.77	3.83	5.23	6.47	5.82	25.24	5.04	74.76
Sugar	5.23	6.24	5.15	5.75	6.27	6.83	6.7	6.91	5.09	8.96	5.58	5.87	5.48	19.92	80.08
To	78.15	71.99	76.23	88.86	78.8	103.48	95.05	94.91	59.01	91.96	71.64	83.63	74.65	69.88	1138.22
Inc.Own	100.15	93.33	96.06	105.25	94.71	119.28	109.83	109.3	76.02	109.94	94.81	101.63	99.89	89.79	TCI = 92%
NET	0.15	−6.67	−3.94	5.25	−5.29	19.28	9.83	9.3	−23.98	9.94	−5.19	1.63	−0.11	−10.21	87.56
NPT	7	4	6	7	5	13	10	9	0	11	3	7	6	3	

Notes: Each cell denotes the amount of connectedness/spillovers from the commodity recorded in the column to the commodity recorded in the row. The column ‘FROM others’ depicts the spillovers from all other variables to each row variable. The row ‘TO others’ depicts the spillovers from each column variable to all other variables. The row ‘Inc. own’ depicts the spillovers from each column variable to all variables, including itself. The row ‘NET’ captures the net connectedness, where a positive (negative) value shows a shock transmitter (receiver).

**Table 2b:** Average Dynamic Connectedness (using QVAR having 0.5 Quantile).

	Cocoa	Coffee	Tea	Coconut oil	Groundnut oil	Palm Oil	Soybeans	Maize	Rice	Wheat	Orange	Beef	Chicken	Sugar	FROM
Cocoa	59.65	4.85	4.15	2.14	2.39	3.72	6.45	3.16	2.37	5.06	2.1	1.22	1.68	1.07	40.35
Coffee	6.23	59.12	3.41	3.29	1.04	5.5	5.58	4.15	1.01	4.05	1.76	1.17	1.15	2.53	40.88
Tea	1.69	5.66	61.98	3.22	1.5	3.48	8.09	2.82	0.66	2.39	2.34	2.24	3	0.93	38.02
Coconut oil	6.94	3.55	1.91	37.62	1.6	19.39	6.58	5.42	1.56	6.45	2.5	2.3	1.64	2.53	62.38
Groundnut oil	3.09	1.46	3.72	4.56	43.56	11.89	6.99	4.71	2.44	4.96	5.77	1.65	2.32	2.9	56.44
Palm Oil	5.74	4.92	2.49	12.11	3.53	36.66	9.9	7.05	0.95	6.58	4.26	2.42	1.57	1.81	63.34
Soybeans	3.03	2	2.8	4.4	2.06	10	40.14	12.89	2.79	10.63	2.45	2.16	2.63	2.01	59.86
Maize	1.51	2.46	1.89	4.03	1.59	8.31	10.96	35.79	2.08	20.41	2.92	3.3	2.14	2.61	64.21
Rice	1.29	1.43	2.36	3.88	1.62	4.54	2.34	1.52	68.67	4.63	1.56	2.26	1.05	2.86	31.33
Wheat	1.33	3.47	2.64	4.34	1.2	7.55	7.29	8.49	3.43	51.72	2.46	3.38	1.11	1.57	48.28
Orange	2.04	1	2.27	1.76	2.75	6.16	3	1.86	2.63	2.2	67.73	2.74	2.75	1.1	32.27
Beef	4.33	5.85	2.77	4.73	1.8	6.2	4.25	2.55	1.63	3.76	4.78	52.01	3.69	1.66	47.99
Chicken	2.26	1.44	1.56	1.88	1.26	3.53	6.14	5.19	2.05	1.2	6.2	4.03	61.61	1.66	38.39
Sugar	3.31	4.73	4.02	1.48	1.4	2.64	5.54	2.35	1.24	9.07	1.74	2.38	1.64	58.48	41.52
To	42.8	42.82	35.98	51.82	23.74	92.9	83.13	62.16	24.85	81.38	40.85	31.24	26.37	25.23	665.27
Inc.Own	102.45	101.93	97.96	89.44	67.31	129.56	123.27	97.95	93.51	133.1	108.58	83.25	87.98	83.7	TCI = 58%
NET	2.45	1.93	−2.04	−10.56	−32.69	29.56	23.27	−2.05	−6.49	33.1	8.58	−16.75	−12.02	−16.3	51.17
NPT	7	7	6	5	1	12	9	7	6	10	10	4	2	5	

Notes: Each cell denotes the amount of connectedness/spillovers from the commodityt recorded in the column to the commodity recorded in the row. The column ‘FROM others’ depicts the spillovers from all other variables to each row variable. The row ‘TO others’ depicts the spillovers from each column variable to all other variables. The row ‘Inc. own’ depicts the spillovers from each column variable to all variables, including itself. The row ‘NET’ captures the net connectedness, where a positive (negative) value shows a shock transmitter (receiver).

**Table 2c:** Average Dynamic Connectedness (using QVAR having 0.9 Quantile).

	Cocoa	Coffee	Tea	Coconut oil	Groundnut oil	Palm Oil	Soybeans	Maize	Rice	Wheat	Orange	Beef	Chicken	Sugar	FROM
Cocoa	16.95	8.46	6.93	5.55	5.62	7.03	7.71	6.86	4.51	8.33	5.31	5.14	4.6	7	83.05
Coffee	8.42	19	5.73	6.17	4.7	6.07	12.64	4.75	3.55	6.22	4.97	4.78	5.5	7.49	81
Tea	6.81	7.95	16.29	7.7	5.69	6.66	9.24	5.63	5.7	5.12	5.59	5.41	4.98	7.25	83.71
Coconut oil	6.59	7.25	7.69	11.5	4.85	8.46	12.94	6.69	5.02	5.85	5.43	5.45	5.61	6.68	88.5
Groundnut oil	6.74	6.13	6.13	8.06	10.66	8.28	11.36	8.2	4.65	7.78	4.35	4.82	5.79	7.05	89.34
Palm Oil	6.96	8.15	7.9	8.71	5.05	10.41	12.2	7.59	4.17	7.47	4.67	4.79	5.96	5.97	89.59
Soybeans	6.19	5.56	6.35	6.77	5.31	7.84	19.87	8.36	4.45	6.36	4.84	4.95	5.69	7.46	80.13
Maize	5.55	5.68	5.76	6.24	4.49	7.53	12.99	12.67	5.08	9.97	5.23	5.38	5.55	7.86	87.33
Rice	5.95	5.21	6.28	6.19	6.83	7.17	14.66	6.25	12.93	6.9	4.17	4.33	6.03	7.11	87.07
Wheat	5.67	5.97	4.7	5.75	3.97	7.12	12.65	9.16	5.49	13.37	6.45	5.03	7.07	7.62	86.63
Orange	6.36	5.53	6.47	5.46	4.47	5.78	8.77	5.87	5.86	5.37	18.44	7.76	6.77	7.09	81.56
Beef	6.77	6.7	5.83	6.13	5.17	6.86	11.28	6.42	5.7	5.69	5.79	14.78	6.02	6.85	85.22
Chicken	4.91	4.95	6.21	6.83	4.9	6.97	9.35	9.04	5.39	5.04	5.17	7.07	15.6	8.57	84.4
Sugar	5.44	5.06	5.24	5.59	5.6	8.48	6.2	5.78	6.41	8.6	6	5.8	4.31	21.5	78.5
To	82.34	82.59	81.21	85.15	66.65	94.25	142	90.61	65.97	88.7	67.96	70.71	73.86	94.01	1186.02
Inc.Own	99.29	101.59	97.5	96.65	77.31	104.67	161.87	103.29	78.89	102.07	86.4	85.5	89.46	115.51	TCI = 93%
NET	−0.71	1.59	−2.5	−3.35	−22.69	4.67	61.87	3.29	−21.11	2.07	−13.6	−14.5	−10.54	15.51	91.23
NPT	6	9	8	7	3	7	12	8	2	10	1	2	5	11	

Notes: Each cell denotes the amount of connectedness/spillovers from the commodity recorded in the column to the commodity recorded in the row. The column ‘FROM others’ depicts the spillovers from all other variables to each row variable. The row ‘TO others’ depicts the spillovers from each column variable to all other variables. The row ‘Inc. own’ depicts the spillovers from each column variable to all variables, including itself. The row ‘NET’ captures the net connectedness, where a positive (negative) value indicates a shock transmitter (receiver).

## 5 Concluding remarks and policy implications

More often than not agri commodities are influenced directly by global events of different natures. However, the embedded pattern of their shock (both receiving & emitting) was not researched. This study found that risk spillover/connectedness never came down (from 1962 to 2022) for the fourteen agri commodities under consideration. It is always at a higher level (more than 55%) proving the fact that agri commodities remain vulnerable to various shocks throughout. Moreover, the connectedness/spillover looks symmetric as both the extreme tails enjoy about 92–93% levels, whereas the median is well below 60%. Rice, Orange Juice, Chicken, Tea and Groundnut Oil were consistent net receivers across such a long-time frame, whereas Palm Oil, Soyabeans, Maize and Wheat were net emitters all through. Further, we found decreasing complexity (network connectedness reduction) with increased quantiles. This complexity reduction in absorbing and emitting shocks is relatively consistent at lower quantiles and drops drastically at a significantly higher quantile. This can be a new ‘stylized fact’ that would direct the risk spillover research towards extremely higher quantiles.

Since, these findings are over such an extended period, policy decisions can be made based on them. Our empirical results provide evidence of volatility spillovers in examined agricultural markets, which has received a little attention in the literature. The commodities we examine in our paper are some of the major agri commodities that concern consumers and producers. Thus, those commodities are essential for policy makers in the agricultural sector, whose responsibility is to introduce regulations and policies in order to reform prices and subsidies. Climate change mitigation policies and adaptation, unexpected weather conditions, crises like COVID-19 or Russia-Ukraine conflict, the influence of global macroeconomic and political strategies with regards energy, re-direction of R&D, add to future agricultural production, price and trade uncertainties.

Policymakers can do, and some already are doing, things to reduce the uncertainties with the above issues. For example, trade negotiations and agreements via the WTO, since trade opening can lead to a more effective use of the limited resources and reduce the existing poverty, inequality and vulnerability of agri commodities. Risks related to fluctuating prices can also be mitigated through the use of several market-based options, such as future markets and insurances. However, these instruments are often rather costly in terms of fees and prices. Some countries attempted to increase their support for these instruments in the last years, but agricultural insurances are still immature and deviate widely amongst countries. Additionally, government authorities could commit to a more determined program for agricultural R&D investment. Farmers should be encouraged to use new technologies to increase their efficiency. Future studies can use advanced econometric methods to examine the potential impact of hypothetical shocks on agri commodities markets.

## Author contribution statement

Bikramaditya Ghosh: Conceived and designed the experiments; Performed the experiments; Analyzed and interpreted the data; Contributed reagents, materials, analysis tools or data; Wrote the paper. DR DIMITRIOS PAPARAS: Conceived and designed the experiments; Wrote the paper.

## Funding statement

This research did not receive any specific grant from funding agencies in the public, commercial, or not-for-profit sectors.

## Data availability statement

Data will be made available on request.

## Declaration of competing interest

The authors declare no competing interests.
